# PERSPECTIVES: Stroke survivors' views on the design of an early‐phase cell therapy trial for patients with chronic ischaemic stroke

**DOI:** 10.1111/hex.12932

**Published:** 2019-07-23

**Authors:** Anjali Nagpal, Susan Hillier, Austin G. Milton, Monica A. Hamilton‐Bruce, Simon A. Koblar

**Affiliations:** ^1^ Stroke Research Programme, Adelaide Medical School, The University of Adelaide South Australian Health & Medical Research Institute (SAHMRI) Adelaide SA Australia; ^2^ Sansom Institute for Health Research University of South Australia Adelaide SA Australia; ^3^ Stroke Research Programme, Departments of Neurology and Medicine The Queen Elizabeth Hospital, & Royal Adelaide Hospital, Central Adelaide Local Health Network (CALHN) Adelaide SA Australia

**Keywords:** informed consent, patient participation, qualitative research, stem cell research, stroke, survivors

## Abstract

**Background:**

Stem cell research holds the potential for a paradigm shift in the management of diseases such as stroke. Patient and public involvement in research (PPIR) can bring a focus to issues of clinical relevance and accelerate translation to real‐world clinical practice.

**Objective:**

A qualitative thematic analysis of the perspectives of stroke survivors regarding the conduct and design aspects of a proposed phase I clinical cell therapy study in stroke.

**Design:**

Twelve stroke survivors were purposively recruited in July 2016–August 2017 and participated in semi‐structured, face‐to‐face interviews for input into the design of a proposed phase I clinical study of autologous dental pulp stem cells. Concurrent thematic analysis was conducted until data saturation was achieved.

**Discussion and conclusions:**

Participants conveyed that the most relevant outcomes to them were regaining participation, decreased dependence on caregivers and improvement in cognition, memory, mood, pain and fatigue. The perception of risk vs. benefit was likely influenced by the time elapsed since stroke, with participants being more willing to accept a higher level of risk early in the post‐stroke disease course. They believed that all stroke survivors should be given an opportunity to participate in research, irrespective of their cognitive capacity. A relatively small sample population of 12 stroke survivors was studied as thematic saturation was achieved. PERSPECTIVES study applied principles of PPIR to early‐phase cell research. Incorporation of outcomes relevant to patients' need within the study design is critical to generate data that will enable personalized application of regenerative medicine in stroke.

## 1 INTRODUCTION

The Global Burden of Disease Study (2015) reported that stroke was the second highest cause of years of life lost globally.^1^ While the age‐standardized mortality from ischaemic stroke in the past decade has declined, approximately 33 million ischaemic stroke survivors worldwide continue to experience lifelong disability and nearly 80% of patients with ischaemic stroke return home with residual impairment.^1^

Regenerative medicine represents a paradigm shift in approach to disease management with the possibility of potential cure or long‐lasting remission for many disease conditions with high‐unmet need. Early clinical studies with stem cell therapies support a novel approach for neuroregeneration and repair following stroke with a potentially longer window of opportunity.^2^ Cell therapy comprises a composite of different cell types being investigated in different phases of stroke, with use of different dose and delivery regimens.

Clinical translation in stroke has been riddled with a disappointing failure of numerous promising preclinical therapeutic candidates, over the last few decades. Currently available therapies are limited in application to the acute phase of stroke.^3^ Stroke represents a diverse set of disease trajectories defined by distinct temporal patterns of neurovascular injury unique to a given patient.^4^ The heterogeneity in patient and disease characteristics has contributed to challenges in choosing the appropriate trial design as well as population and efficacy parameters. This, in turn, makes it difficult to assess the effect size of an intervention and its clinical relevance and validity.

Recognition of patients as key partners/stakeholders in research has been increasing over the past decade. It represents a promising approach to generate evidence that is relevant and trustworthy for patients and their families as well as clinicians.^5^ This is likely to contribute to a greater sense of empowered participation in patients who are the eventual users of the outcomes of such research.^6^

Research evidence reporting facilitators and barriers to clinical trial participation and patient experiences with clinical research is increasing, particularly in areas such as cancer and stroke.^7^ Studies have investigated the relative importance of issues regarding research, in people with stroke.^8^, ^9^ These reported that there was a discrepancy between priorities and relevance attributed to different outcomes by different stakeholders in research, such as patients, caregivers and researchers.^8^, ^9^

Early patient engagement is likely to be associated with increased recruitment and retention of study participants; development of research methods that are contextualized to patients' experiences with the disease; and utilization of relevant research questions and outcome measures. There is growing evidence for the value of ‘patient and public involvement in research’ (PPIR) in facilitating more patient‐focused research by offering insights into prioritization, design and implementation and making trials more effective and credible.^10^ PPIR is increasingly being mandated for publicly funded trials in many developed countries.^11^

Active participation of potential participants is likely to provide a sense of empowerment to people with chronic stroke. Their engagement as ‘lay experts’ to provide their perspective on clinical relevance of different aspects of study design is likely to improve the eventual study design. The increase in transparency and credibility of research associated with such partnership is specifically critical in innovative areas of research such as stem cell therapies. This was a key issue raised by stroke survivors who participated in the Stroke Survivors' Forum held at the South Australian Health and Medical Research Institute (SAHMRI), in Adelaide in 2014.

In response to this advice, the PERSPECTIVES study sought to formatively collect insight into the beliefs and perspectives of people with chronic stroke through their involvement in the design of the TOOTH study (The Open study Of dental pulp stem cell (DPSC) Therapy in Humans). The TOOTH study aimed to investigate the effectiveness of autologous administration of adult dental pulp stem cells in people with chronic ischaemic stroke.^12^

The study aimed to explore the views of people with chronic stroke on:

- the relevance and importance of an early phase clinical study such as TOOTH, with adult human dental pulp stem cells in chronic ischaemic stroke
- the relevance and acceptability of the planned outcome measures and study design of the TOOTH study and
- issues with consent to participate in the TOOTH study.

## 2 METHODS

### 2.1 Study design

The study involved a naturalistic design, adapting from a participatory action‐research approach to explore stroke survivors' perspective on early clinical research design with cell therapy (TOOTH). The study methodology fits within a constructionist epistemology paradigm, utilizing an inductive thematic analysis.

### 2.2 Ethics approval

Ethics approval for conducting this study was granted by the Human Research Ethics Committees of the University of Adelaide (Ref: H‐2016‐089) and the University of South Australia (Ref: 0000035776).

### 2.3 Study population, sampling and participant recruitment

The study recruited people with chronic ischaemic stroke who were residents of Adelaide and likely to fulfil the proposed selection criteria for participation in the TOOTH trial as listed below:

#### 2.3.1 Inclusion criteria

- Inclusion of both genders.
- Age of the participant 18 years or over.
- History of chronic ischaemic stroke with a stable level of disability.
- Sufficient cognitive and language ability to participate in an interview.

#### 2.3.2 Exclusion criteria

- Impaired cognition or significant psychological issues.
- Inability to communicate in the absence of a caregiver.
- Inability to travel to the interview location.

### 2.4 Study enrolment

Eligible participants were recruited using purposive sampling from the research database of people with stroke, maintained by the Stroke and Rehabilitation Research Group (SRR) in the Sansom Institute for Health Research, University of South Australia. The SRR database includes individuals who are periodically followed in the course of their post‐stroke rehabilitation. Those individuals from the database who were known to be sufficiently competent cognitively to engage in an interview involving moderate complexity, had no prior established diagnosis of depression or other significant psychological issues and had indicated their consent to be contacted for future research were invited to participate (SH). Participants were enrolled on an on‐going basis during the period: July 2016 to August 2017, until the concurrent thematic analysis suggested that data saturation had been achieved.

Following an expression of interest in participation, all potential study participants received a participant information pack containing the participant information sheet for the PERSPECTIVES study and the summary information sheet on the TOOTH trial which described the study design and current understanding of the benefit and risk, given that this is a first‐in‐human safety study. AN followed up with the participants by telephone to address any queries regarding the study information provided and obtain verbal consent to participate. Following written informed consent, the individuals participated in a semi‐structured interview at SAHMRI. The interview was conducted in line with key areas of enquiry defined in the interview guide (Table 1), regarding the research design of the TOOTH trial. The subquestions were adapted to lines of response provided by the participant. All interviews were audio‐recorded and professionally transcribed verbatim by OutScribe Pty Ltd, Adelaide, South Australia.

**Table 1:** Interview guide: Key areas of inquiry

Interview: Topic guide
What has been the impact of stroke on your daily life?
What are your views on using stem cell therapies for managing stroke?
What are your thoughts on the usefulness of a study like TOOTH?
What are the effects that are important to measure in a study like TOOTH?
Are there any specific risks that you feel should be measured in the TOOTH?
Would it be appropriate for participants with impaired thinking or understanding to participate in a study like TOOTH?

### 2.5 Data analysis

Analysis of audio transcripts was carried out immediately after every interview, and data were coded by AN using NVivo software Version 11 (QSR International). AN read and reread the transcripts and constructed an index of multiple emerging codes. This index was discussed and cross‐checked with SK, SH and AHB. Coding was an iterative process that proceeded concurrently with on‐going interviews. As new codes were added, previous transcripts were recoded to further refine the coding framework analysis of the data.^13^ The inductive thematic analysis continued until no new code emerged from subsequent interviews; that is, data saturation was achieved. Subsequent analysis crystallized the key themes that represented the aspects emerging from refining of codes from the data.

## 3 RESULTS

### 3.1 Participant disposition

SH contacted 31 patients with stroke following review of their functional status. Following their indication of interest in participation, they were provided with study information by AN. Nineteen patients declined to participate, mostly due to time or mobility constraints. Twelve patients participated in face‐to‐face interviews, following provision of written consent. Patients were asked to complete a patient profile to understand the overall impact of stroke on their lives along with their age and stroke latency. The population was diverse concerning these parameters, but representative of the potential target population for stroke trials (Table 2).

**Table 2:** Study participants' characteristics

Age	Range = 42‐81 y	
Time since stroke	Range = 0.5‐14 y	
Impact of stroke on activities of daily living and ability to function independently	VAS* Scale	No. of participants
	1‐5	3
	6‐10	9
Interest in participation in TOOTH	Response	No. of participants
	Yes	7
	No	2
	Not sure	3

* Visual Analogue Scale of 1‐10:1 being no/minimal impact to 10 being significant impact.

Data saturation was achieved after twelve interviews with no new themes emerging after eight interviews.

### 3.2 Themes

**Figure 1:**
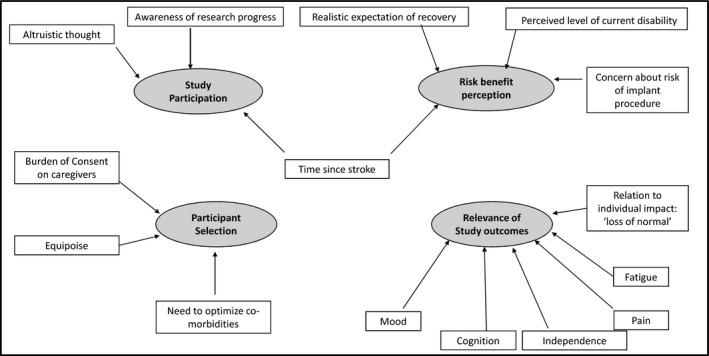
Key themes

The themes described below represent themes identified, even though some elements may overlap (Figure 1).

#### 3.2.1 Real‐life relevance of study outcomes—Are all equally meaningful?

The participants conveyed that the most meaningful change for them would be a change in their ability to participate in life interactions and their ability to get back to doing activities of interest, that is, ‘being more normal’ with lessened dependence on significant others (caregivers) in their lives.

Participant 001: I can't play my music, I can't sew, I can't knit, I can't sing anymore. It's changed it a lot. I guess I would like the use of the things I've said that I can't do.

Participant 002: Oh just being human. Just being able to get up in the morning and do the things by yourself, without needing help.

Participant 008: It affects your personal life and you're sitting there like an inanimate lump and so it has an adverse effect on your intimacy too.

They conveyed that tests that can measure and track changes over time, in the impairments specific to a given patient, would be more meaningful to pursue as markers of therapy benefit.

Participant 005: Whilst I understand why there are certain tests that you do at the beginning of therapy, and at the end of the therapy range, I guess that understanding a person's day to day life and how they do things, and being conscious of it, what improvements it could mean to the individual.

Participant 008: So putting pegs on a board and things like that. If they can measure that, and they should be, they've got the measuring devices now, say you got 40% one day, six months later you got 45%, that'd be brilliant.

Patients reported subtle impairment in terms of the difficulty encountered in processing information and new learning situations. This was critical for patients as it jeopardized their ability to effectively participate in social and work‐related interactions.

This in turn decreased their sense of self‐worth and contributed to feeling depressed, as reported by the participants. They reported that this adversely impacted the quality of their life after stroke.

Participant 002: And basically that stage, because you can't, can't do your normal things. And basically, everything, every, little things add up… call it frustration, call it what you want, but the more things don't go right for you, the worse you become. Yeah and it happens every day.

Measuring the impact of cell therapy on mood changes would be useful to monitor in any prospective study evaluating their effectiveness.

Participant 002: Yep. If they can help that out, that would be a major step, seriously. Yep. Because I reckon, I reckon your figure would be up over 90% of people who get very depressed. And depression leads to sort of not wanting to do normal things. And of course, while you're not doing them, your body’s shutting down even further, so.

The interviewees also expressed their interest in measuring change in pain and fatigue, which they associated with a significantly adverse quality of life experienced post‐stroke.

Participant 005: I've met so many people who've had strokes … but I think the only real common theme is the fatigue and, possibly, the pain.

While improvement in speech was acknowledged as a relevant outcome to be measured, the issue of interest was the impact of this impairment on ease of communication and confidence in social interactions.

#### 3.2.2 Risk‐benefit‐perception

Interviewees were quite pragmatic in their expectation of recovery of function, accepting that full recovery to pre‐stroke level of functioning may not be achievable.

Participants believed that perhaps it was not realistic to expect complete recovery of function and they would consider any positive change in functional ability to be meaningful.

Participant 006: Obviously, I would love to have the use of everything perfect again, but I know that's probably something that's not going to happen. But just, for me, just not to have the pain so much.

The interviewees accepted that potential safety issues could be expected, given the early stage of the research. The perception of risk was consistent across the participant group. Participants expressed concern regarding risk of further functional impairment.

Participant 010: I would worry that I could end up, worse off. That something unforeseen could happen and, maybe another part of my brain could die off.

Participants also expressed concerns with the transplantation procedure, related to the extent of hospital stay required and risk of complications (eg infections) associated with the procedure. Interestingly, these concerns were related to risk associated with the surgical procedure per se and not with the issue of stem cell implantation under MRI guidance.

Death or cancer derived from cell transplantation was not cited as the most critical concerns, which is interesting, given that these adverse events are considered the most important events to investigate and report by the research community. However, participants expressed their desire to know about any available information with regard to cancer risk.

#### 3.2.3 Attitude towards trial participation

The interviewees expressed varied interest in participation ranging from no interest, to unsure, to a very keen interest to participate—often this related to their perceived level of current disability.

Participant 002: Basically I got my body as good as it's going to get. If I had stem cells put in me, and something went wrong, and it brought me back even 5%, then I've done all those years for nothing. But if I got offered stem cells right at the start, I would’ve thought, yeah go for it.

The perception of possible benefit and the associated willingness to participate was impacted by the time since stroke irrespective of the extent of present disability. Participants expressed that their willingness to participate in studies such as TOOTH would have been higher ‘earlier in their disease’ course and defined that period to be within the first year following the stroke event. The common driver of this sentiment appears to be an apprehension of losing the ‘gains’ made with existing management strategies, because of engaging in an investigational treatment.

Participants conveyed having very limited knowledge about stem cell research, particularly in the field of stroke, even though quite a few of them were aware of research with stem cells for other diseases such as Parkinson disease and multiple sclerosis.

Participant 005: I've heard about stem cell research, with the likes of Parkinson's, and other heart problems, etc. I haven't heard of anything regarding stroke.

Interviewees expressed an opinion that the proposed TOOTH study was relevant for people with disability following stroke. The predominant driver of this belief seemed to be an altruistic thought process regarding this research contributing to a potentially beneficial therapy to answer a current lack of meaningful therapy options and consistent rehabilitation.

#### 3.2.4 Attitude towards participant selection

Participants communicated that study participation should, in principle, be available to any patient with existing disability and that it was inappropriate to exclude patients with cognitive incapacity. Proxy consent in such situations may be deemed acceptable but only in situations where consent was provided by a close member of the family, who would likely be aware of the patient's wishes and likely preferences. However, such consent should not be provided by a professional caregiver. They recognized that proxy consent represents a significant psychological burden on the carers.

Participant 002: I'd like to see everybody involved in the study, it's probably an ethical thing. But in the case of (carer), if you’ve got full trust in the carer, yes, but, I mean that’s putting a lot on the individual carer. I mean if it is a family person, something like that, it's probably better. Like, someone of course had to care for me for a while and all that sort of stuff.

Participants expressed the view that it was important to ensure that relevant rehabilitation and secondary prevention strategies such as control of hypertension, lipid levels and weight are optimized for any patient selected in the study, as these are likely to influence the eventual study outcome.

## 4 DISCUSSION

### 4.1 Key findings and implications for research

The unique challenges posed by personalized application of stem cell therapies pose a question of whether the research designs can be optimized to facilitate meaningful progress in this field.^2^

This study reports on the views and perspectives of ischaemic stroke survivors on the study design of a proposed Phase 1 open‐label cell therapy trial. The study explores the key outcomes (effectiveness and safety) of interest to them. The insights generated are likely to have an important impact on the design of such proposed studies and to be a useful reference to guide trial design for similar stem cell studies in the future. Importantly, it highlights that active participation of patients in research design can result in trials that generate data, which are more meaningful for the patient community. This provides additional specific evidence to support the value of PPIR in optimizing research quality.

The critical focus of early phase research has always been to gather evidence for safety while establishing proof of concept. In the context of stem cell therapies, it is universally accepted that early‐phase studies in healthy volunteers is ethically unacceptable.^14^ This presents an interesting opportunity to tap into lived experiences of stroke in patients to optimize research design and produce clinically relevant data that enables efficient decision making in clinical practice.^15^ Patient involvement in early phase research is likely to help researchers understand what potential safety concerns are important/relevant to them.^16^, ^17^ Our study reports that patients are more concerned about the risk of losing their current level of functioning, rather than the potential risk listed in study materials, based on preclinical and postulated biological mechanisms, such as tumorigenicity, or conventional risks such as mortality. This highlights the need for researchers to ensure that research data specifically address this identified patient need as well as clinician and legal requirements.

Early clinical studies seek to collect exploratory evidence of benefit. The heterogeneity in presentation and recovery trajectory of stroke has always presented a challenge in terms of defining study designs that can generate scientifically rigorous yet clinically meaningful efficacy data.^17^ Our study indicates that the patient community has a significant depth of insight about this conundrum. Stroke survivors suggested that the selection criteria of patients should include optimization of secondary prevention strategy and patient‐specific rehabilitation strategies. Emerging research postulates that the recovery trajectory in most patients can be predicted based on existing integrity of neural pathways and current level of brain atrophy.^18^ It might be interesting to consider whether the suggestion by our community for optimization of post‐stroke care is an intuitive exercise in enrichment for ‘responders’ on the proportional recovery prediction rule, thereby selecting individuals that are likely to have most benefit with additional investigational interventions. Finally, patients suggested that measurement of change in clinical outcomes needs to be personalized to patient‐specific impairment. Using this approach can potentially lead to identification of homogenous patient clusters, which may enable a more efficient assessment of effect size and appropriate target population.

A continuing debate in the research and clinician community is whether the outcome measures currently utilized are valid and relevant to the patients' life‐experience following stroke.^19^, ^20^ The National Institutes of Health Stroke Scale (NIHSS) is an established measure of stroke severity and literature supports its use in the prognosis of post‐stroke recovery.^21^ The Modified Rankin Score (mRS) is the most commonly selected primary endpoint in drug and rehabilitation studies.^22^ While it assesses a range of outcomes, including severe disability and death, it is not adequately sensitive to assess cognition, mood or return to social and occupational functioning.^19^ The use of these endpoints on their own to define success or failure of studies, particularly those involving personalized therapy options such as stem cell therapies, may not adequately measure the range of outcomes found to be critical from a patient perspective. The World Health Organization (WHO) proposed the International Classification of Functioning (ICF)^23^ and recommend outcomes evaluation within dimensions of body function impairments, activity limitations and participation restrictions. In the context of stroke, a very small percentage of pharmacological or rehabilitation studies have to date examined impact on participation restriction, as is also the case for cognition and mood outcomes.^19^ Evidence for widespread prevalence of issues in these domains reported by patients has been steadily increasing in recent years.^20^ Research involving patient‐reported outcomes has described persistent and significant impact on patients' lives even for those that fully recover their pre‐stroke functional level. The present study reiterates the importance of these outcomes and their measurement to the patients. The International Consortium for Health Outcomes Measurement (ICHOM) conducted an iterative Delphi process that included diverse stakeholders such as clinicians, patients, stroke registers and stroke societies.^24^ The study suggested a ‘Stroke Standard Set’—a minimum dataset of outcomes and risk adjustment variables to collect for all patients hospitalized with stroke. The categories recommended within the ICHOM standard set for assessment were survival and disease control, acute complications, and patient‐reported outcomes (PROM). PROM included assessment at 90 days for pain, mood, feeding, self‐care, mobility, communication, cognitive functioning, social participation, ability to return to usual activities, and health‐related quality of life, along with data on mobility, feeding, self‐care, and communication, collected at discharge. Collecting data on these parameters using validated tools at different phases of stroke targeted in early clinical studies would build the quantum of data available on the magnitude of effect that is plausible with cell therapies. The increased understanding of anticipated effect can better inform decisions on minimal clinically important difference (MCID) that are acceptable and meaningful to clinical practice. It stands to reason that such informed decision making would contribute to increased efficiency in later phases of development and more informed and relevant study size determination and design.

Participants indicated an acceptance of their current level of functioning and that the return of functioning over time was due to consistent effort on their part to engage with the rehabilitation options available. This drove the heightened concern for the potential risk of loss of this functional improvement that they worked very hard to achieve. They indicated that they would have been more accepting of this potential risk if an opportunity to participate in a clinical study such as TOOTH had arisen ‘earlier’ in their disease course, defined as within a year of their stroke occurrence. This insight has important implications for study recruitment for stem cell studies in chronic stroke. A recently reported study also highlighted these as important determinants of interest in participation.^25^ While the time of CT administration is likely to be still be determined by the existing understanding of mechanistic properties of the investigational treatment from preclinical studies, allowing for stabilization of patient's general medical condition and spontaneous post‐stroke recovery to take place are reasonable postulates.^26^ However, this may present an ethical challenge in instances where data on mechanisms of action indicate benefit in acute/subacute phase of stroke. Our study suggests that targeting a narrower window for recruitment (up to 1 year after the stroke event) might facilitate recruitment and provide a more favourable risk‐benefit proposition to potential participants. Most researchers in stem cell research in stroke accept that stem cell therapies in practice are likely to be codelivered with rehabilitation.^27^ Application of stem cell therapies at a time point following stroke where rehabilitation has achieved maximum possible benefit may enable clearer distinction of incremental change with stem cell therapies.

A long‐standing debate in stroke trials is the issue of consent particularly for patients with cognitive deficit.^28^ Our results report the opinions of individuals interviewed, who expressed that opportunity to participate should be equitably available to all, regardless of their cognitive capacity. Proxy consent by next of kin is now well accepted in the context of acute trials in stroke.^8^ Participants indicated that proxy consent might be considered in stem cell studies, even in the chronic phase of stroke. However, they highlighted that this was likely to place a significant psychological burden on the caregivers and acknowledged the practical challenges involved in the process. Cunningham et al^25^ reported similar findings as a potential ‘care conflict’ between patients and caregivers in such research situations. It is pertinent to state that challenges with consent remain particularly relevant and complicated, both at the initial consent to participate and on‐going consent through the course of study conduct.

Our study reports low levels of awareness about on‐going research with stem cell therapies in stroke and limited understanding of postulates regarding their mechanism of action. A recent study by Aked et al^29^ also reported similar findings regarding the low level of awareness regarding stem cell research. Patient advocacy groups constitute a promising though still underutilized means of increasing this awareness. The participants in our study, who reported prior knowledge of stem cells, attributed this to information they received from such groups. Patient involvement in research from an early phase can also help build awareness and knowledge in the stroke patient community. This is likely to enable patients to become more informed and empowered participants in research.

In a broader societal and medical practice context, the increased awareness of current state of regenerative neurology in stroke and evidence‐based estimation of risk and benefit to be expected can become a deterrent to unscrupulous use of unproven stem cell therapies for commercial use and stem cell tourism.

### 4.2 Strengths and limitations

The study utilized face‐to‐face interviews with stroke survivors, as the method of qualitative enquiry. This enabled a relaxed and supportive environment in which they shared their individual preferences, contextualized to their unique lived experience with stroke. The study findings highlighted key outcomes considered important from patient perspectives and that need to be measured within study design.

This approach also minimized the dilution of information likely with other modalities such as combined focused groups with other stakeholders such as caregivers. Our study is a part of a wider exercise that will also explore views from different stakeholders, particularly caregivers, in separate studies. The rationale behind this strategy is based on the growing body of evidence for disconnect in the perception/acceptance of risk and benefit, between patients and caregivers.^15^, ^17^, ^25^, ^30^, ^31^

The requirement to travel and engage in an in‐depth interview meant that the study did not include patients with very severe disability, cognitive deficits and severe aphasia. The participants were therefore not fully representative of the overall stroke survivor community. However, the perspectives shared by them in the context of preferences for study design components were largely agnostic to the degree of severity of post‐stroke disability and may well be more relevant to the broader group. The severity of present disability has been shown previously to impact on motivation to participate in other studies,^8^, ^29^ and this was corroborated in our study. However, it did not appear to influence the relative importance assigned to different outcome and design elements. The intention of our study was to provide rich thematic description of our qualitative enquiry. Previous studies with similar research methodology have reported this to be possible, though recruiting large number of patients was found to be challenging.^32^, ^33^, ^34^ The eventual number of participants may appear rather small. However, the research team conducted constant comparison of emerging themes to ensure that data saturation was confirmed to ensure validity of study findings.

## 5 CONCLUSIONS

The PERSPECTIVES study applied principles of patient and public involvement in research in early clinical stem cell research in stroke. Engagement of stroke survivors as ‘lay experts’ to provide input into study designs can provide critical insights that can enable more targeted research. In an evolving field such as cell therapy in stroke, this partnership can potentially help researchers to efficiently address the challenges posed by the inherently ‘personalized’ field of regenerative medicine.

## CONFLICT OF INTEREST

The authors declare no conflicts of interest.

## AUTHORS' CONTRIBUTIONS

AN involved in ethics application, study design and conduct (participant consent/ recruitment/interviews), data collection, analysis and manuscript preparation. SH involved in supervision of study design and conduct, provided access to the patient database for study recruitment and critical review of data analysis and manuscript. AGM involved in ethics application and critical review of manuscript. MAHB and SAK involved in supervision of study design and conduct and critical review of data analysis and manuscript.

## ETHICAL APPROVAL

Ethics approval for conducting this study was granted by the Human Research Ethics Committees of the University of Adelaide (Ref: H‐2016‐089) and the University of South Australia (Ref: 0000035776).

## DATA SHARING

The data that support the findings of this study are available on request from the corresponding author. The data are not publicly available due to privacy restrictions.

## References

[1] Feigin VL , Forouzanfar MH , Krishnamurthi R , et al. Global and regional burden of stroke during 1990–2010: findings from the Global Burden of Disease Study 2010. Lancet. 2014;383(9913):245‐255. 24449944 10.1016/s0140-6736(13)61953-4 PMC4181600

[2] Nagpal A , Choy FC , Howell S , et al. Safety and effectiveness of stem cell therapies in early‐phase clinical trials in stroke: a systematic review and meta‐analysis. Stem Cell Res Ther. 2017;8(1):191. 28854961 10.1186/s13287-017-0643-x PMC5577822

[3] Tymianski M . Novel approaches to neuroprotection trials in acute ischemic stroke. Stroke. 2013;44(10):2942‐2950. 24021680 10.1161/STROKEAHA.113.000731

[4] Hossmann KA . The two pathophysiologies of focal brain ischemia: implications for translational stroke research. J Cereb Blood Flow Metab. 2012;32(7):1310‐1316. 22234335 10.1038/jcbfm.2011.186 PMC3390813

[5] Sheridan S , Schrandt S , Forsythe L , Hilliard TS , Paez KA . The PCORI engagement rubric: promising practices for partnering in research. Ann Fam Med. 2017;15(2):165‐170. 28289118 10.1370/afm.2042 PMC5348236

[6] Gamble C , Dudley L , Allam A , et al. Patient and public involvement in the early stages of clinical trial development: a systematic cohort investigation. BMJ Open. 2014;4(7):e005234. 10.1136/bmjopen-2014-005234 PMC4120322 25056972

[7] Snape D , Kirkham J , Britten N , et al. Exploring perceived barriers, drivers, impacts and the need for evaluation of public involvement in health and social care research: a modified Delphi study. BMJ Open. 2014;4(6):e004943. 10.1136/bmjopen-2014-004943 PMC4067891 24939808

[8] Ali K , Roffe C , Crome P . What patients want consumer involvement in the design of a randomized controlled trial of routine oxygen supplementation after acute stroke. Stroke. 2006;37(3):865‐871. 16456122 10.1161/01.STR.0000204053.36966.80

[9] Saywell N , Taylor D . Focus group insights assist trial design for stroke telerehabilitation: a qualitative study. Physiother Theory Pract. 2015;31(3):160‐165. 25404160 10.3109/09593985.2014.982234

[10] Gamble C , Dudley L , Allam A , et al. An evidence base to optimise methods for involving patient and public contributors in clinical trials: a mixed‐methods study. Health Serv Deliv Res. 2015;3(39):1‐142. 26378330

[11] Evans TW . Best research for best health: a new national health research strategy. Clin Med (Northfield Il.). 2006;6(5):435‐437. 10.7861/clinmedicine.6-5-435 PMC4953958 17080885

[12] Nagpal A , Kremer KL , Hamilton‐Bruce MA , et al. TOOTH (The Open study Of dental pulp cell therapy in Humans): Study protocol for evaluating safety and feasibility of autologous human adult dental pulp cell therapy in patients with chronic disability after stroke. Int J Stroke. 2016;11(5):575‐585. 27030504 10.1177/1747493016641111

[13] Braun V , Clarke V . Using thematic analysis in psychology. Qual Res Psychol. 2006;3(2):77‐101.

[14] Food and Drug Administration , Considerations for the Design of Early‐Phase Clinical Trials of Cellular and Gene Therapy Products, U.S. Department of Health and Human Services. 2015 http://www.fda.gov/downloads/BiologicsBloodVaccines/GuidanceComplianceRegulatoryInformation/Guidances/CellularandGeneTherapy/UCM359073.pdf: accessed 12th January, 2016.

[15] Harrison M , Palmer R . Exploring patient and public involvement in stroke research: a qualitative study. Disabil Rehabil. 2015;37(23):2174‐2183. 25598139 10.3109/09638288.2014.1001525

[16] Sacristán JA . Clinical research and medical care: towards effective and complete integration. BMC Med Res Methodol. 2015;15(1):1‐7. 25575454 10.1186/1471-2288-15-4 PMC4323129

[17] Solomon NA , Glick HA , Russo CJ , Lee J , Schulman KA . Patient preferences for stroke outcomes. Stroke. 1994;25(9):1721‐1725. 8073449 10.1161/01.str.25.9.1721

[18] Winters C , van Wegen EE , Daffertshofer A , Kwakkel G . Generalizability of the proportional recovery model for the upper extremity after an ischemic stroke. Neurorehabil Neural Repair. 2015;29(7):614‐622. 25505223 10.1177/1545968314562115

[19] Kapoor A , Lanctôt KL , Bayley M , et al. "Good Outcome" isn't good enough: cognitive impairment, depressive symptoms, and social restrictions in physically recovered stroke patients. Stroke. 2017;48(6):1688‐1690. 28438907 10.1161/STROKEAHA.117.016728

[20] Gray J , Lie ML , Murtagh MJ , Ford GA , McMeekin P , Thomson RG . Health state descriptions to elicit stroke values: do they reflect patient experience of stroke? BMC Health Serv Res. 2014;14:573. 25413030 10.1186/s12913-014-0573-6 PMC4254212

[21] Rost NS , Bottle A , Lee J‐M , et al. Stroke severity is a crucial predictor of outcome: an international prospective validation study. J Am Heart Assoc. 2016;5(1):e002433. 26796252 10.1161/JAHA.115.002433 PMC4859362

[22] Chaisinanunkul N , Adeoye O , Lewis RJ , et al. Adopting a patient‐centered approach to primary outcome analysis of acute stroke trials using a utility‐weighted modified rankin scale. Stroke. 2015;46(8):2238‐2243. 26138130 10.1161/STROKEAHA.114.008547 PMC4519373

[23] Kostanjsek N Use of The International Classification of Functioning, Disability and Health (ICF) as a conceptual framework and common language for disability statistics and health information systems. BMC public health. 2011;11(Suppl 4), S3. 10.1186/1471-2458-11-S4-S3 PMC3104216 21624189

[24] Salinas J , Sprinkhuizen SM , Ackerson T , et al. An international standard set of patient‐centered outcome measures after stroke. Stroke. 2016;47(1):180‐186. 26604251 10.1161/STROKEAHA.115.010898 PMC4689178

[25] Cunningham NA , Abhyankar P , Cowie J , Galinsky J , Methven K . Regenerative medicine: Stroke survivor and carer views and motivations towards a proposed stem cell clinical trial using placebo neurosurgery. Health Expect. 2018;21(1):367‐378. 29024214 10.1111/hex.12632 PMC5750757

[26] Langhorne P , Bernhardt J , Kwakkel G . Stroke rehabilitation. Lancet. 2011;377(9778):1693‐1702. 21571152 10.1016/S0140-6736(11)60325-5

[27] Kalladka D , Muir KW . Brain repair: Cell therapy in stroke. Stem Cells Cloning. 2014;7(1):31‐44. 24627643 10.2147/SCCAA.S38003 PMC3937183

[28] Rose DZ , Kasner SE . Informed consent: the rate‐limiting step in acute stroke trials. Front Neurol. 2011;2:65. 22022320 10.3389/fneur.2011.00065 PMC3195267

[29] Aked J , Delavaran H , Lindvall O , Norrving B , Kokaia Z , Lindgren A . Attitudes to cell therapy among ischemic stroke survivors in the lund stroke recovery study. Stem Cells Dev. 2017;26(8):566‐572. 28142330 10.1089/scd.2016.0343

[30] Visser‐Meily A , Post M , Gorter JW , Berlekom S , Van Den Bos T , Lindeman E . Rehabilitation of stroke patients needs a family‐centred approach. Disabil Rehabil. 2006;28(24):1557‐1561. 17178619 10.1080/09638280600648215

[31] Dudley L , Gamble C , Preston J , et al. What difference does patient and public involvement make and what are its pathways to impact? Qualitative study of patients and researchers from a cohort of randomised clinical trials. PLoS ONE. 2015;10(6):e0128817. 26053063 10.1371/journal.pone.0128817 PMC4459695

[32] Bennett L , Luker J , English C , Hillier S . Stroke survivors' perspectives on two novel models of inpatient rehabilitation: seven‐day a week individual therapy or five‐day a week circuit class therapy. Disabil Rehabil. 2016;38(14):1397‐1406. 26600073 10.3109/09638288.2015.1103788

[33] Baker SE , Edwards R , Doidge M . How many qualitative interviews is enough?: Expert voices and early career reflections on sampling and cases in qualitative research. 2012.

[34] Guest G , Bunce A , Johnson L . How many interviews are enough? An experiment with data saturation and variability. Field Methods. 2006;18(1):59‐82.

